# Effects of education to facilitate knowledge about chronic pain for adults: a systematic review with meta-analysis

**DOI:** 10.1186/s13643-015-0120-5

**Published:** 2015-10-01

**Authors:** Louise J. Geneen, Denis J. Martin, Nicola Adams, Clare Clarke, Martin Dunbar, Derek Jones, Paul McNamee, Pat Schofield, Blair H. Smith

**Affiliations:** Division of Population Health Sciences, University of Dundee, Dundee, UK; Institute of Health and Social Care, Teesside University, Middlesbrough, UK; Department of Sport, Exercise and Rehabilitation, University of Northumbria, Northumbria, UK; Division of Cardiovascular and Diabetes Medicine, University of Dundee, Dundee, UK; NHS GG&C/University of Glasgow, Glasgow, UK; DipCOT, Independent Researcher, Edinburgh, UK; Institute of Applied Health Sciences, University of Aberdeen, Aberdeen, UK; Centre for Positive Ageing, School of Health and Social Care, University of Greenwich, Greenwich, UK

**Keywords:** Education, Chronic pain, Physical function, Disability, Catastrophising

## Abstract

**Background:**

Chronic pain can contribute to disability, depression, anxiety, sleep disturbances, poor quality of life and increased health care costs, with close to 20 % of the adult population in Europe reporting chronic pain. To empower the person to self-manage, it is advocated that education and training about the nature of pain and its effects and how to live with pain is provided. The objective of this review is to determine the level of evidence for education to facilitate knowledge about chronic pain, delivered as a stand-alone intervention for adults, to reduce pain and disability.

**Methods:**

We identified randomised controlled trials of educational intervention for chronic pain by searching CENTRAL, MEDLINE, EMBASE and ongoing trials registries (inception to December 2013). Main inclusion criteria were (1) pain >3 months; (2) study design that allowed isolation of effects of education and (3) measures of pain or disability. Two reviewers independently screened and appraised each study.

**Results:**

Nine studies were analysed. Pooled data from five studies, where the comparator group was usual care, showed no improvement in pain or disability. In the other four studies, comparing different types of education, there was no evidence for an improvement in pain; although, there was evidence (from one study) of a decrease in disability with a particular form of education—pain neurophysiology education (PNE). Post-hoc analysis of psychosocial outcomes reported in the studies showed evidence of a reduction in catastrophising and an increase of knowledge about pain following PNE.

**Conclusions:**

The evidence base is limited by the small numbers of studies, their relatively small sample sizes, and the diversity in types of education studied. From that limited evidence, the only support for this type of education is for PNE, though it is insufficiently strong to recommend conclusively that PNE should be delivered as a stand-alone intervention.

It therefore remains sensible to recommend that education be delivered *in conjunction* with other pain management approaches as we cannot confidently conclude that education alone is effective in reducing pain intensity or related disability in chronic pain in adults.

**Electronic supplementary material:**

The online version of this article (doi:10.1186/s13643-015-0120-5) contains supplementary material, which is available to authorized users.

## Background

Chronic pain can contribute to disability, depression and anxiety, sleep disturbances, poor quality of life, and increased health care costs, with close to 20 % of the adult population in Europe reporting chronic pain [1].

Chronic pain is recognised as a long-term condition in its own right or a major comorbidity of other long-term conditions. An aim of the management of all long-term conditions, embodied in the idea of self-management, is that the patient should be an active participant in the management of their own condition. In chronic pain management, such thinking is based on the assertion that “self-care and management underpins all activities in the care pathway and should be considered [at all stages]… pain management is most effective when it engages the patient in self-management” [2]. To empower the person to self-manage, it is advocated that education and training is provided about the nature of pain and its effects and how to live with pain. Responding to recommendations to establish what educational interventions in pain management work best and for whom [3] is complex not least because of the many different methods and combinations of methods of education and training that are available and in use.

The intervention being investigated in this study is education of the patient to facilitate their knowledge of chronic pain that does not include behaviour modification or training in skills of pain management.

Whilst the use of other non-pharmacological interventions can generally be informed by good quality systematic reviews of the literature (e.g. cognitive behavioural and behavioural therapy [4–6], TENS [7] and low-intensity movement therapy [6, 8]), that level of evidence is less readily available to inform the use of education to facilitate knowledge about chronic pain in adults.

Therefore, a systematic review of available studies that have evaluated the effectiveness of education to facilitate knowledge about chronic pain in adults was conducted to assess the current situation and inform future research and guidelines.

### Review objectives

The primary objective was to determine the current level of evidence of the effect of education to facilitate knowledge about chronic pain for adults on pain and disability. A secondary objective was to determine (from the selected papers) the evidence of effect of the educational interventions on psychosocial outcomes.

## Methods

### Inclusion/exclusion criteria

#### Study type

Suitable for inclusion were randomised controlled trials (RCTs) and cluster-RCTs published and pre-published (electronically) in peer-reviewed journals; studies were accepted as randomised when described by the author as such. Studies were included if they were published in English. Studies were excluded if they were only available in abstract form.

#### Type of participants

Studies of adults (aged 18 years or older) reporting pain for at least 3 months (12 weeks) at any body-site(s) were included. Mixed age samples were included if data could be separated (adult/child). We excluded studies that were focused on specific diagnoses (e.g. osteoarthritis) and conditions where chronic pain is not necessarily the main symptom (e.g. irritable bowel syndrome), as the advice or guidance was likely to be based on the specific impact and management of the condition (e.g. joint stiffness, bowel dysfunction) at least as much as on the management of pain. We also excluded cancer-related pain.

We only included studies that focused on chronic pain, which is consistent with a strategy used in previous national guidelines [6].

#### Interventions

Studies were included where the effectiveness of education to facilitate knowledge about chronic pain could be assessed as a stand-alone intervention compared with usual care, or where different types of education could be compared with each other. Therefore, we excluded investigations of multi-disciplinary programmes (such as pain management programmes) which did not allow assessment of the effectiveness of the education component.

#### Outcome measures

Studies were included if they reported outcomes of pain severity and/or physical function.

### Data collection and analysis

#### Electronic searches

We searched EBSCOhost [MEDLINE, CINAHL Plus, OmniFile (Full text), eBook Collection] and CENTRAL [Cochrane Central Register of Controlled Trials; MEDLINE, EMBASE, Cochrane Review Groups Specialist Registers] databases from inception to 31 December 2013. No language restrictions were imposed whilst searching; English language criterion was applied later in the process. The search strategy was developed for use in MEDLINE and adapted for each database (Additional file 1).

#### Other resources

Reference lists of reviews and the 22 articles reviewed in detail by all of the authors (see below) were checked for additional studies, and citation searches were performed on key articles to minimise publication bias.

Ongoing trials were searched using the World Health Organization International Clinical Trials Registry Platform (ICTRP) (http://apps.who.int/trialsearch/) and the metaRegister of Controlled Trials (mRCT) (http://www.controlledtrials.com/), which includes the ISRCTN Register (international), Action Medical Research (UK), NIH ClinicalTrials.gov Register (international), the Wellcome Trust (UK) and UK trials (UK).

#### Selection of studies

Following initial scanning of titles, abstracts and full papers by one author, two authors read the remaining full papers and assessed them separately. Authors independently selected studies that met the inclusion criteria using a purpose-designed checklist, limiting inclusion to studies that were randomised (or cluster-randomised) as a minimum. Further discussion was required when the authors could not reach a consensus on the studies to be included. Authors with psychological expertise reviewed the studies to remove those deemed to have formally implemented an underlying psychological therapy as part of, or including, education to facilitate knowledge about chronic pain. In this manner, 22 studies were included after screening and were then assessed by all authors. Finally, those studies that fulfilled all of the inclusion criteria were selected for analysis, a total of nine.

#### Data extraction and management

Data were extracted using a standardised form which included information regarding study design, participants, trial characteristics, intervention, comparison (control) and outcomes. Data were collected manually on paper extraction forms and entered into intermediate software (Microsoft Excel for Windows) before being entered in to RevMan 5.3 [9]. This intermediary stage allowed for any necessary statistical conversions. Only one pain measure was selected per study. When there were multiple measures of pain in a study, we used only the measure of average pain intensity. When there was more than one report of average pain intensity, then the mean of these was calculated. Results from visual analogues scores (VAS) were prioritised over measures such as the McGill Pain Questionnaire (MPQ) if both were reported.

#### Risk of bias assessment

Two authors independently assessed risk of bias for each study. Arbitration by a third author was not necessary as inconsistencies were resolved through discussion. The domain-based evaluation presented in the *Cochrane Handbook for Systematic Reviews of Interventions* (Chapter 8, version 5.1.0 [10]) was used to assess risk of bias. We assessed the following for each study: random sequence generation (checking for possible selection bias), allocation concealment (selection bias), blinding of patients and personnel (performance bias), blinding of outcome assessment (detection bias), incomplete outcome data (attrition bias due to the amount, nature and handling of incomplete outcome data) and selective reporting (reporting bias). For “other” sources of bias, we included criteria to evaluate study sample size, where fewer than 50 participants per treatment arm was considered an increased risk of bias.

Risk of bias across all included studies was categorised according to the following:

- Low risk of bias (✓) – plausible bias unlikely to seriously alter the results if most information was obtained from studies at low risk of bias
- Unclear risk of bias (?) – plausible bias that raised some doubt about the results if most information was obtained from studies at low or unclear risk of bias
- High risk of bias (X) – plausible bias that seriously weakens confidence in the results if the proportion of information was obtained from studies at high risk of bias sufficient to affect interpretation of results

#### Measurement of treatment effect

Data from included studies were reviewed separately and then, where possible, combined quantitatively by population, intervention, comparison and outcomes. Continuous data were expressed as mean difference (MD) or standardised mean difference (SMD) with 95 % confidence intervals (95 % CI); dichotomous data were reported by just one study [11] for a single outcome measure (session attendance), and this finding has been reported in the results of this review as text only.

#### Assessment of heterogeneity

We assessed heterogeneity according to the standard method using the Chi [2] test and the *I* [2] statistic, calculated for each comparison on each outcome. *I* [2] values above 50 % suggest high heterogeneity, 25–50 % medium heterogeneity and below 25 % low heterogeneity, though this is only used as a guide.

A standard random effects analysis was used to avoid over-weighting large studies and potentially losing small study effects.

#### Assessment of reporting bias

We intended to use funnel plots to assess small-study effects, following the guidance of the *Cochrane Handbook of Systematic Reviews for Interventions* (Chapter 10) [10], but studies were insufficient in number (*n* = 9) to undertake this effectively.

#### Data synthesis

Data were entered into RevMan 5.3 [9] by one author and checked by a second. Data were largely presented as mean and standard deviation (SD); though one study [11] reported results as mean and standard error (SE), these data were converted to mean and standard deviation using the RevMan calculator, and results were checked by hand. One study reported only average (mean) results with no measure of variation [12], and we were unable to extract plausible data for inclusion in the meta-analyses or other form of data presentation within this review.

#### Analysis

Studies with a comparator group of usual care were analysed separately from the studies that compared different types of education. The issues with aging and pain are more complex than simply pain being a direct correlate of biological age, though there does appear to be justification for considering pain in older people as a distinct issue [13–16]. Therefore, where data on older people (>65 years) could be extracted, these were analysed separately. Sensitivity analysis was also planned to determine if the effect size was affected by the methodological quality of the study (risk of bias). However, these analyses were not possible due to the small number of included studies.

Using the same approach as above, we also carried out post-hoc analyses of the following psychosocial variables which were reported in these studies: catastrophising, mood, knowledge of chronic pain, self-efficacy, global health and social function.

## Results

### Search results

**Fig. 1:**
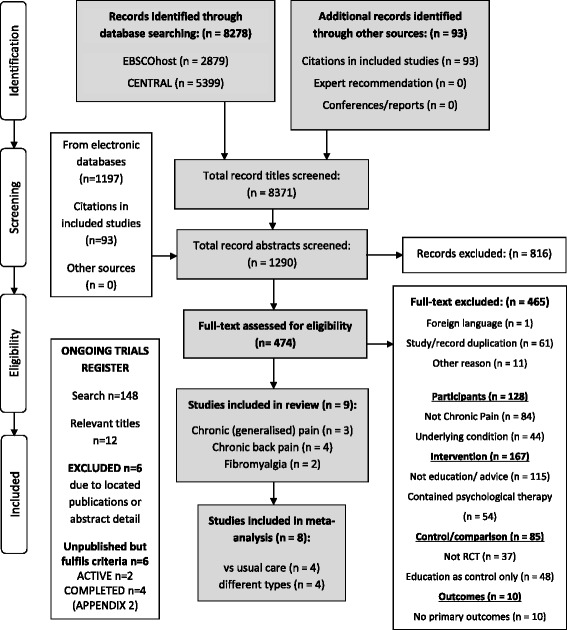
PRISMA [43] flow chart demonstrating database searches, identification, screening and selection of included studies

Results of the search are shown in Fig. 1. Summary information of the studies included in the review is shown in Table 1.

**Table 1:** Characteristics of Included studies

Author (year) in chronological order	Specialty	Patient eligibility and recruitment	Trial characteristics	Participant characteristics	Intervention and follow-up periods	Outcome measures reported
Studies assessing education versus usual care						
Linton et al. 1997. Orebro (Sweden)	Chronic back pain	Age 18-60yrs old, accumulated sick leave for MSK pain of 2–24 weeks in the past year. Recruited via screening of insurance files, and through adverts in local newspaper	Parallel design, three arms (two interventions: “Educational support”, and “Professional support”, one control - we are not including “Professional support”). “Educational support”: patient-based support group with education, insight and empathy, used *mastering pain* self-help book. “Control”: regular treatment group, no additional effort to facilitate or prohibit.	“Educational support” *n* = 39 (74 % F), pain duration average 26 months; “Control” *n* = 25 (68 % F), pain duration average 26 months	“Educational support”: met for 180 min 15 times in 1 year; once/week for a month, every 2 weeks for 3 months, every other month for 5 months. Outcome measures at baseline and 1 year later	Sickness impact profile (SIP-pain), 50-item coping strategies questionnaire (CSQ), multidimensional pain inventory (MPI). Attendance, outcome evaluation questionnaire, sick leave from work. three pain beliefs and attitudes: pain and impairment relationship scale (PAIRS), Pain and discomfort scale (PADS), pain beliefs and perceptions inventory (PBPI)
Soares et al. 2002. Stockholm (Sweden)	Fibromyalgia	FM diagnosed in previous 2 years, female, 18–64 years, no other serious illness, no ongoing drug/alcohol abuse, not involved in other therapies. Recruited via GPs working in Stockholm area.	Parallel design, three arms (two interventions: “educational intervention” and “behavioural intervention”, one control - we are not including behavioural intervention). “educational intervention”: focus was on information about various health related topics inc the body, FM, pain, sleep hygiene, medication, managing crises, ergonomic education, self-management	All female, “educational intervention” *n* = 18, mean age 47 years, pain duration 50 months, “wait list control” *n* = 17 mean age 43 years, pain duration 37 months	“Education intervention”: two individual sessions (2 h each) and 15 group sessions (2 h each, 3–5 patients per group) for 10 weeks (total 102 h). Outcome measures at baseline, post-intervention, and 6 months later.	The pain questionnaire (PQ), the arthritis self-efficacy scale (ASES), The McGill Pain Questionnaire (MPQ), The coping strategies Questionnaire (CSQ), The Karolinska Sleep Questionnaire (KSQ), “The Diary” VAS-pain The Fibromyalgia Impact Questionnaire (FIQ), The symptom checklist - revised (SCL-90-R), The Interview Schedule of Social Support (ISSI),
Ruehlman et al. 2012. Arizona (USA)	Chronic pain	Over 18 yrs old, chronic pain for 6 months or more, access to computer with high speed internet, English language fluency. Recruitment via online pain sites.	Parallel design, two arms (intervention: online Chronic Pain Management Program, control: wait-list/usual care). “CPMP” has four learning modules of both online and offline activities (e.g. didactic and interactive material online, homework and self-monitoring offline), includes social networking component.	total *n* = 305 (196 F, 109 M), age 19–78 years mean 45 years, pain over 2 yrs in 90 % of sample, “CPMP” *n* = 162, “control” *n* = 143	“CPMP” unsupervised access to website for 6 weeks i.e. self-directed and self-paced online program. Outcome measures at baseline, 7 weeks (i.e. post-intervention period), and at 14 weeks.	Profile of Pain: Screen (PCP-S), Centre for Epidemiological Studies Depression Scale (CES-D), Depression Anxiety and Stress Scale (DASS), pain knowledge, attitudes and beliefs: profile of pain extended assessment (PCP-EA), pain interference in functioning
Sparkes et al. 2012. Cardiff (UK)	Low back pain	Over 18yrs old, LBP with or without referral to the lower limbs, referred to spinal pain clinic by GP, English language fluency. Recruited via referrals sent to the spinal pain clinic.	Parallel design, two arms (intervention: The Back Book, control: usual care/wait list control).	“Back Book” *n* = 29 (13 M, 16 F) mean age 52 years, “control” *n* = 28 (11 M, 17 F) mean age 52 years	“Back Book” posted a copy of the book whilst waiting to be seen by specialist as part of referral process. Asked not to read until they had completed initial questionnaires. No follow-up letters sent which may have encourage compliance. Outcome measures at baseline (posted questionnaires after screening for inclusion/exclusion), and follow-up (at patients’ initial SPC consultation).	VAS-pain Back Beliefs Questionnaire (BBQ), Fear-avoidance beliefs questionnaire - physical activity (FABQ -PA), Roland Morris Disability Questionnaire (RMDQ), VAS-understanding of the back book
Excluded from analysis due to inability to extract data (Morrison et al. 1988)						
Morrison et al. 1988. British Colombia (Canada)	chronic back pain	All (non-adolescent) patients routinely admitted to the back pain program between November 1981 and May 1982 participated. Referrals made by GPs and specialists.	Not a classic design—used sequential instead of concurrent assessment. Each group only assessed once. Attempted to strengthen study by repeated time sampling (collecting data for six different sets of patients, each with their own control group)	Mean age 45 years (range 17–74 years), *n* = 120 (63 % F), no individual group stats	six 3-h sessions over 2-, 3- or 6-week period: lectures and demonstrations of anatomy, physiology, body mechanics, posture, stress recognition and management, pain relief, physical exercise, and first aid techniques. Each group assessed only once; control at baseline, intervention group at the end. One year after completion, a random sample of intervention-ers (*n* = 28) re-assessed (physical function), and (*n* = 85) returned follow-up questionnaires	Oswestry Pain Scale (OPS), education - use of correct body mechanics, and patient knowledge (15-item quiz). State Anxiety Inventory (SAI). Function - strength and mobility, self-reported exercise, RAND physical abilities scale
Author (year) in chronological order	Specialty	Patient eligibility and recruitment	Trial characteristics	Participant characteristics	Intervention and follow-up periods	Outcome measures reported
Ferrell et al. 1997. California (USA)	chronic musculo-skeletal pain	Over 65 years old, presence of lower extremity pain, use of analgesics, ambulatory without assistance, English language fluency. Recruited from a Veterans Admin Medical Centre in response to info brochure mailed to home address	Parallel design, three arms (two intervention: “physical methods” and “walkers”, one control - we are not including the “walkers” intervention). “physical methods”: 90minute education session of non-drug interventions. “control”: attention control, received printed material with general info about pain and management	Mean age 73 years, “physical methods” *n* = 10 (3 F, 7 M), pain duration 1–53 years; “control” *n* = 10 (10M), pain duration 10 months–53 years	Two orientation sessions prior to intervention to educate about pain. ”physical methods” one-off education session. Outcomes measures at baseline (pre-randomisation; t1), following the two orientation sessions (t2), and 6 weeks later (t3)	Patient Pain Questionnaire (PPQ), RAND 36-item health Survey (SF-36). three performance tests: 6 min walk test, sit to stand 30, sit and reach test
Moseley et al. 2004. Brisbane and Sydney (Australia)	Chronic low back pain	Primary reason for presentation at pain clinic was LBP longer than 6 months. Recruited by A note advertising the project was included in the material given to patients at three private rehab clinics.	Parallel design, two arms (intervention: neurophysiology education, control: back education)	Intervention: “neurophysiology education” *n* = 31 (13 M, 18 F), mean age 42 years, pain duration average 29 months. Control “back education” *n* = 27 (12 M, 15 F), mean age 45 years, pain duration average 30 months	Both groups same format: took part in a one-off education session in 1-to-1 seminar format, each session was 3 h long with a 20 min break. Homework was a workbook in ten sections - read one section, answer three questions each weekday for 2 weeks.	Compliance. Roland Morris Disability Questionnaire (RMDQ). Survey of Pain Attitudes, revised (SOPA-R). Three physical performance measures - straight leg raise (SLR), forward bending range, abdominal drawing in task (ADIT), Pain Catastrophising Scale (PCS)
Gallagher et al. 2013. Adelaide and Sydney (Australia)	Chronic pain	Age 18–75 years, pain that disrupts ADLs for more than previous 3 months, English language fluency. Recruited from waiting list for multidisciplinary pain management program.	Partial (control group) cross-over design, two arms (intervention: “book of metaphors to help understand the biology of pain”, control: “advice about managing pain”, then crossed over to intervention). “book of metaphors” each section was a short story, followed by interpretation. “control/ advice booklet” each section focussed on a concept of pain management and drew heavily from the back book and manage your pain.	“metaphors” *n* = 40 (26 F, 14 M), age = 42 years, pain duration =25 months. “advice/control” *n* = 39 (22 F, 17 M), age = 45 years, pain duration = 31 months	Both groups received information in the same format—booklet of 80 pages in 11 sections. Outcome measures at baseline, and emailed questionnaires 3 weeks later, and two months after that (“12 weeks”).	Pain assessed on 11-point numerical rating scale (NRS), pain biology questionnaire (PBQ), pain catastrophising scale (PCS), disability/function—Five tasks assessed on 11-point numerical rating scale (NRS)
Van Oosterwijck et al. 2013. Brussels (Belgium) and Glasgow (UK)	Fibromyalgia	Age 18–65 years, FM defined by the criteria of the 1990ACR, have Dutch as native language	Parallel design, two arms (intervention: neurophysiology education, control: activity management education)	Intervention “neurophysiology” *n* = 15 (3 M, 12 F) age = 46 years, symptom duration = 156 months. Control “activity management” *n* = 15 (1 M, 14 F) age = 46 years, symptom onset = 116 months	2 one-on-one education sessions. Intervention and control differed in content only. First session used powerpoint presentation of 30 min. Leaflet handed out. Second session 1 week later delivered by telephone. Outcome measures at baseline (pre), 2 weeks (post), and 3 months (follow-up). Additional outcome measure (PPT and neurophysiology questionnaire) also tested after first education session.	Spatial summation procedure (SSP), Health status survey (SF36), pain coping inventory (PCI), Pain Vigilance and Awareness Questionnaire (PVAQ), Tampa Scale Kinaesiophobia (TSK), pressure pain threshold (PPT) pain catastrophising scale (PCS), fibromyalgia impact questionnaire (FIQ), Neurophysiology of pain test

Searches for *ongoing* trials revealed 148 trials, of which twelve titles fulfilled the inclusion criteria. From these, six were excluded after further examination of the abstract/trial registration detail or due to duplication with previously located studies. The remaining six studies have potential to be included in future updates but are currently unavailable to this review (Additional file 2).

### Description of studies

Nine studies were included (Ferrell et al. 1997 [17], Gallagher et al. 2013 [18]; Linton et al. 1997 [11], Morrison et al. 1988 [12], Moseley et al. 2004 [19], Ruehlman et al. 2012 [20], Soares et al. 2002 [21], Sparkes et al. 2012 [22] and van Oosterwijck et al. 2013 [23]). In each study, all of the participants reported pain for at least 3 months. Two studies were conducted on people with fibromyalgia [21, 23], three were on people with chronic pain or chronic musculoskeletal pain [17–19], and four studies specifically examined back pain [11, 12, 19, 22]. Individual study sample size ranged from 20 [17] to 305 [20]. Participants were over the age of 18 years in all studies, though one did not specify this in the criterion [19]. There was no upper age limit in five studies [12, 17, 19, 20, 22], one of which used a sample that was exclusively over 65 years old [17]. Others excluded individuals over the age of 60 [11], 65 [21, 23] and 75 years [18].

All studies except one [23] reported the process of recruiting, which was largely through a general practitioner (GP) or specialist referrals and pain programme waiting lists. Trials were conducted in Canada [12], USA [17, 20], Sweden [11, 21], Australia [18, 19], UK [22] and in both Belgium and the UK [23].

Education to facilitate knowledge about chronic pain was in the form of lectures [12, 17, 19, 23], individual or group discussions [11, 21], written text [18, 22] or website interaction [20]. These interventions took place during a single session [17, 19, 23], numerous interactions (multiple sessions of the same format [11, 12], individual and then group sessions [21]) or with no direct contact [18, 20, 22]. Education varied in focus from understanding the neurophysiology and biology of pain [18, 19, 23] to management of symptoms through accessing physical help, such as medication, hot/cold packs, ergonomics [17, 21] and a combination of these and other topics (e.g. anatomy, physiology, body mechanics, posture, pain relief and first aid techniques [11, 12, 20, 22]).

The comparator group was usual care in five studies [11, 12, 20–22], and the other four studies compared different methods of education [17–19, 23].

The nine studies included in this review reported at least one of the primary outcome measures (Table 4). All studies except one [19] reported an assessment of pain, although there was a variety of measures used for the assessment. Physical function or disability was measured objectively in two studies using validated performance tests [17] and standardised tests for the study [12]. It was also measured subjectively (patient-reported) in a further four studies using a validated disability questionnaire (Roland Morris Disability Questionnaire) [19, 22] and validated assessment of interference or impact on daily life [11, 20].

#### Excluded studies

Thirteen studies were excluded (Table 2). Three of these were excluded due to their multi-disciplinary intervention, where the effect of education alone could not be assessed [24–26]; five were excluded after assessment as having a psychological, rather than an educational content [27–31]; and three more were excluded due to inconsistency with the study design criteria [32–34].

**Table 2:** Excluded studies

Author (year)	Reason for exclusion
Burckhardt et al. 1994	Refers to “a contract for individual behaviour change”, suggesting CBT/BT
Chiauzzi et al. 2010	Second main component of website “CBT to improve self-efficacy”
Dirmaier et al. 2013	Protocol only
Dush et al. 2006	Mentions “psychotherapy components were tailored to patient’s needs”, suggesting psychotherapy in addition to education
Dworkin et al. 2002	Involves relaxation and coping skills training
Haas et al. 2005	Uses Stanford Self-management model (multi-disciplinary, unable to assess educational component alone)
Harpole et al. 2003	Includes detailed clinical assessment and tailored treatment plan
Jerjes et al. 2007	Pilot study, non-randomised
LeFort et al. 1998	Uses Stanford Self-management model (multi-disciplinary, unable to assess educational component alone)
Matchar et al. 2008	Includes diagnosis and treatment as part of the programme
Michelotti et al. 2012	Focus on “habit reversal” (psychological intervention) and includes a large physiotherapy (exercise) component (multi-disciplinary, unable to assess educational component alone)
Van Ittersum et al. 2011	No control group
Vlaeyen et al. 1996	Includes physical exercise at the end of each session, therefore, cannot distinguish effect of education or exercise

### Risk of bias in included studies

Low or unclear/uncertain risk was identified across the majority of the six domains. Risk of bias for each included study is shown in Table 3.

**Table 3:** Risk of bias summary showing the review authors’ judgements about each methodological quality item for each included study

	Selection bias		Performance bias	Detection bias	Attrition bias	Reporting bias	Other bias	Total
Author (year) *In chronological order*	Random sequence generation	Allocation concealment	Blinding of participants and personnel	Blinding of outcome assessment	Incomplete outcome data	Selective reporting	Other (eg. sample size)	No. of low risk of bias (✓)
Morrison et al. 1988	?	?	?	X	?	?	?	0
Ferrell et al. 1997	?	?	?	✓	✓	?	x	2
Linton et al. 1997	✓	✓	?	?	✓	?	?	3
Soares et al. 2002	x	x	✓	✓	✓	?	x	3
Moseley et al. 2004	✓	✓	✓	✓	✓	?	?	5
Ruehlman et al. 2012	?	?	✓	✓	✓	?	✓	4
Sparkes et al. 2012	✓	?	✓	✓	✓	?	?	4
Gallagher et al. 2013	✓	✓	✓	✓	✓	?	✓	6
Van Oosterwijck et al. 2013	✓	✓	✓	✓	✓	?	X	5
No. of studies with low risk of bias - ✓	5	4	6	7	8	0	2	
No. of studies with uncertain/unclear - ?	3	4	3	1	1	9	4	
No. of studies with high risk of bias—X	1	1	0	1	0	0	3	

(✓) is low risk of bias, (X) high risk of bias, (?) unclear or uncertain

#### Selection bias (random sequence generation and allocation concealment)

Four studies fulfilled both criteria for low risk of bias [28, 32, 33, 35], and one fulfilled one of the two criteria [36]. Three studies mentioned that the participants were randomised and allocation-concealed but did not specify the method constituting an unclear risk of bias [29, 31, 34]. One study described itself as randomised, but patients were “consecutively allocated” to each group and so held a high risk of bias [37].

#### Performance bias (blinding of participants and personnel)

All of the most recent studies [32–37] showed low risk of bias and reported blinding of participants and personnel where necessary. Earlier publications [28, 29, 31] did not mention blinding.

#### Detection bias (blinding of outcome assessment)

In five studies, there was no blinding of outcome assessments [31–34, 36], but the review authors judged that the outcome measure was unlikely to be influenced by this knowledge as questionnaires were completed alone by the participant. Two studies reported blinding for outcome measures [35, 37], only one of which reported assessing the success of blinding of both the participants and personnel [35]. The study by Morrison et al. [29] was labelled high risk of bias as each group was only assessed once (the control group at pre-intervention, and treatment group post-intervention only).

#### Attrition bias (incomplete outcome data)

Dropouts and withdrawals were noted and explained in all studies (low risk of bias) except one where there was no mention of incomplete data [29].

#### Reporting bias (selective reporting)

No published protocols were found, and so we cannot say with absolute certainty that all outcome measures were reported, and all included studies were therefore awarded an unclear/uncertain risk of bias.

#### Other potential sources of bias

Study size was assessed as an additional risk of bias, as a small study size could bias the results. The methods specified that fewer than 50 participants per treatment arm would be an increased risk of bias as seen in all but two studies (low risk of bias *n* = 79 after crossover from control [32], *n* = 162 [34]). The remaining seven studies were further separated into those with fewer than 20 participants in the treatment group as high risk of bias in three studies (*n* = 10 [31], *n* = 18 [37], *n* = 15 [35]), uncertain risk for those where *n* ~ 30 in the treatment group [28, 33, 36] and unclear risk for one study [29] that reported no separate treatment/control sample size.

### Intervention effect

1. Average pain intensity (Table 4 post-intervention and Table 5 follow-up).

**Table 4:** Pain and disability outcome measures - post-intervention

Outcome measure	Study	Sample size			Statistic used	Heterogeneity	Effect size		Test for overall effect		Notes
		Intervention	Control	Total		*I* ^2^ (%)		[95 % CI]	*Z*-value	*p*-value	
PAIN INTENSITY											
Education versus usual care											
“average pain”	Linton 1997; Soares 2002; Sparkes 2012; Ruehlman 2013	248	213	461	SMD random	0	−0.01	[−0.19, 0.17]	0.12	0.90	Figure 2
PPQ - pain in the last week	Ferrell 1997	10	10	20	MD random	n/a	−2.80	[−21.09, 15.49]	0.30	0.76	Sample >65 years
Comparison of different types of education											
SF36 - bodily pain	van Oosterwijck 2013	15	15	30	MD random	n/a	−3.40	[19.98, 13.18]	0.40	0.69	
DISABILITY											
Education versus usual care											
Disability or interference	Linton 1997; Ruehlman 2012; Sparkes 2012	230	196	426	SMD random	49	0.02	[−0.31, 0.34]	0.11	0.91	Figure 4
Comparison of different types of education											
SF36 - physical function	van Oosterwijck 2013	15	15	30	MD random	n/a	5.30	[−8.64, 19.24]	0.75	0.46	
Roland Morris Disability Questionnaire	Moseley 2004	31	27	58	MD random	n/a	−2.00	[−3.55, −0.45]	2.53	0.01	Favours education
Function and Disability (pooled data using negative RMDQ score for direct comparison)	van Oosterwijck 2013; Moseley 2004	46	42	88	SMD random	0	0.52	[0.09, 0.95]	2.38	0.02	Figure 6; favours education
SF36 - physical function	Ferrell 1997	10	10	20	MD random	n/a	6.70	[−9.11, 22.51]	0.83	0.41	Sample >65 years

*PPQ* patient pain questionnaire, *SF-36* RAND 36-item health survey, *95 % CI 95 %* confidence interval, effect size represented as standardised mean difference (SMD) or mean difference (MD) depending on statistic used; Random = random effects model; heterogeneity is not applicable (n/a) when reported as single study

**Table 5:** Pain and disability outcome measures - follow-up

Outcome measure	Study	Sample size			Statistic used	Heterogeneity	Effect size		Test for overall effect		Notes
		Intervention	Control	Total		*I* ^2^ (%)		[95 % CI]	*Z-*value	*p*-value	
PAIN INTENSITY											
Education versus usual care											
“average pain”	Soares 2002; Ruehlman 2013	18	17	35	SMD random	0	0.02	[−0.19, 0.24]	0.21	0.83	Figure 3
Comparison of different types of education											
SF36 - bodily pain	van Oosterwijck 2013	15	15	30	MD random	n/a	−9.90	[−24.73, 4.93]	1.31	0.19	
PPQ - pain in the last week	Ferrell 1997	10	10	20	MD random	n/a	−6.50	[−22.94, 9.94]	0.78	0.44	Sample >65 years
DISABILITY											
Education versus usual care											
PCP-S - interference	Ruehlman 2012	162	143	305	MD random	n/a	0.46	[−1.46, 2.38]	0.47	0.64	Figure 5
comparison of different types of education											
SF36 - physical function	van Oosterwijck 2013	15	15	30	MD random	n/a	8.40	[−4.27, 21.07]	1.30	0.19	Figure 7
SF36 - physical function	Ferrell 1997	10	10	20	MD random	n/a	−1.80	[−15.71, 12.11]	0.25	0.80	Sample >65 years

*PCP-S* profile of chronic pain-screening, *PPQ* patient pain questionnaire, *SF-36* RAND 36-item health survey, *95 % CI 95 %* confidence interval; Effect size represented as standardised mean difference (SMD) or mean difference (MD) depending on statistic used; Random = random effects model; heterogeneity is not applicable (n/a) when reported as single study

#### Education versus usual care

*Adults >18 years*: All four studies measured pain at the post-intervention measurement point [11, 20–22], and two of these studies also reported follow-up assessments [20, 21].

**Fig. 2:**
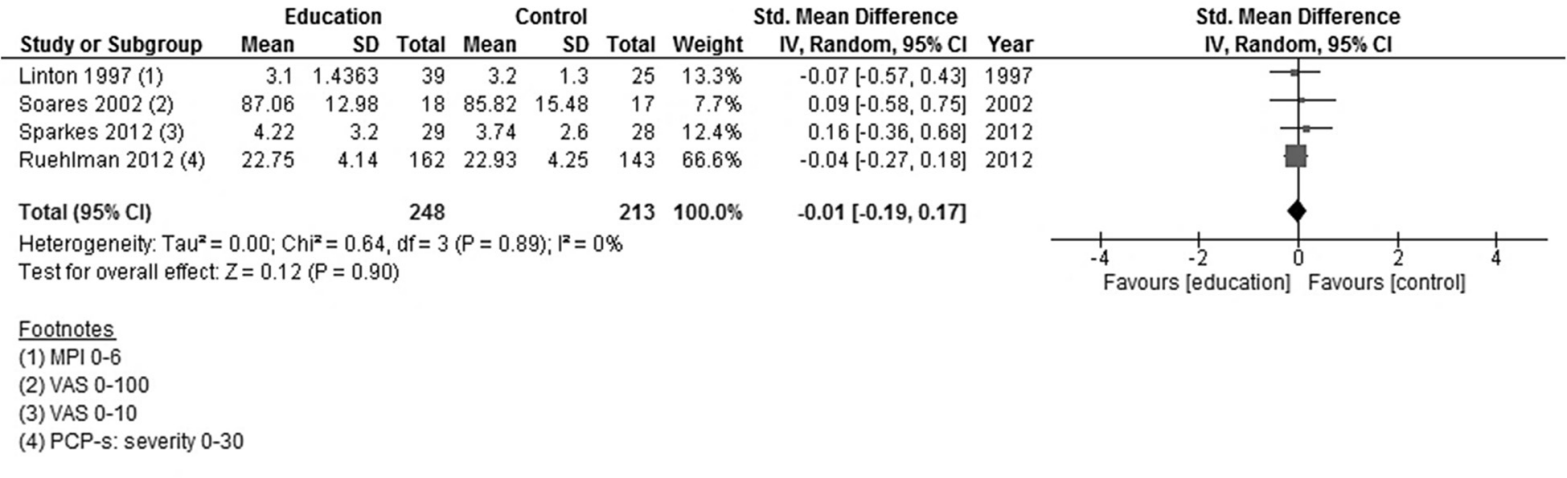
Forest plot showing pain intensity (education versus usual care)—post-intervention

**Fig. 3:**
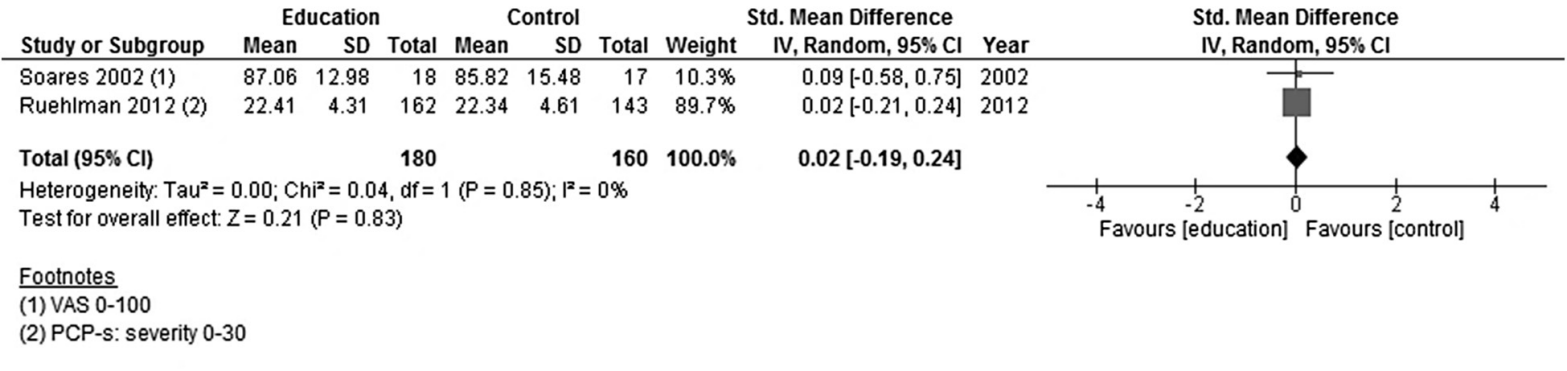
Forest plot showing pain intensity (education versus usual care)—follow-up (3 months)

None of the studies showed significant effects post-intervention. Pooling the data of all four studies showed low heterogeneity (*I* [2] = 0 %), and the effect size was small and statistically non-significant (Fig. 2). In neither of the two studies reporting results around 3 months after the end of the intervention was there a significant effect. Again, pooling of the data showed a small effect size that was not statistically significant (Fig. 3).

*Adults >65 years*: No specific data were available.

#### Comparison of different types of education

*Adults >18 years*: Measures of average pain could only be extracted from one study [23]. In this study, which used a very small sample size to compare pain neurophysiology education (PNE) with another form of information provision, there were no statistically significant differences in average pain intensity between the two forms of information provision 2 weeks after the intervention period or 3 months after the intervention ended.

*Adults >65 years*: One study exclusively investigated adults aged >65 years [17]. The study, which had a very small sample size, found no significant differences between information provision about pain and information provision about physical methods to reduce pain immediately after the intervention period or 6 weeks after completion.

2. Disability (Table 4 post-intervention and Table 5 follow-up).

#### Education versus usual care

*Adults >18 years*: Disability was assessed in three out of the four studies post-intervention [11, 20, 22]. Only one of the studies had a follow-up assessment, reporting results at 3 months from the end of the intervention [20].

**Fig. 4:**
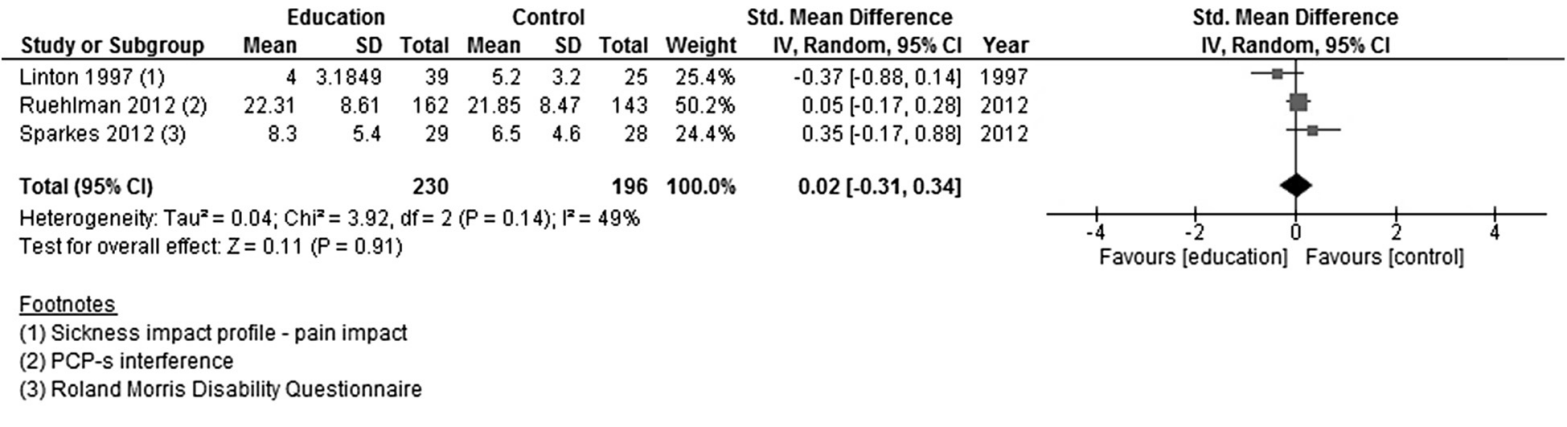
Forest plot showing disability (education versus usual care)—post-intervention

**Fig. 5:**
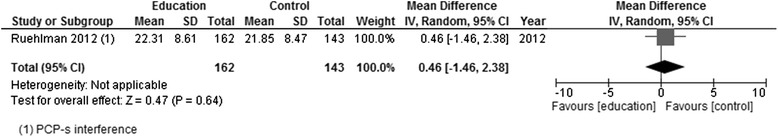
Forest plot showing disability (education versus usual care)—follow-up (3 months)

There were no significant effects on disability in any of the studies immediately after the end of the intervention. When data were pooled, heterogeneity was high amongst these studies (*I* [2] = 49 %), and the overall effect size was low (*Z* = 0.11) and statistically non-significant (Fig. 4). The single study that assessed disability at 3 months following the end of the intervention showed no significant differences in disability between groups (Fig. 5).

*Adults >65 years*: No specific data were available.

#### Comparison of different types of education

*Adults >18 years*: Disability was assessed in two studies, both of which compared pain neurophysiological education (PNE) with other information provision types [19, 23]. Only one study contained a follow-up assessment at 3 months following the end of the intervention; this study used a very small sample size [23].

**Fig. 6:**
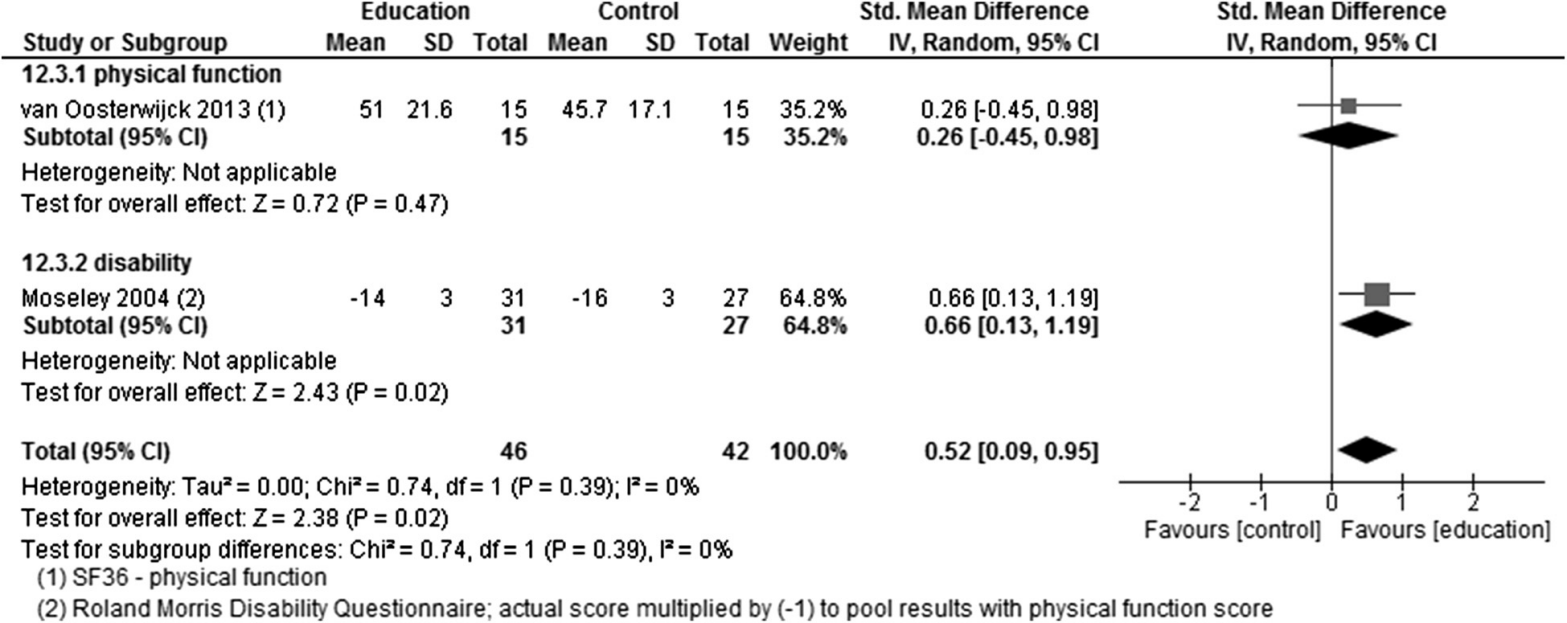
Forest plot showing disability and physical function (comparison of different types of education)—post-intervention

PNE showed a significantly better effect than its comparator on the Roland Morris Disability Questionnaire (RMDQ) scores immediately after the intervention in one study [19]. In the other study, which had a very small sample size [23], the mean difference in favour of PNE (5.3 points on the SF36 physical function subscale 2 weeks after the intervention had ended) was not statistically significant. Pooling the data from both studies showed low heterogeneity (*I* [2] = 0 %), and there was a statistically significant difference in favour of PNE (Fig. 6).

**Fig. 7:**
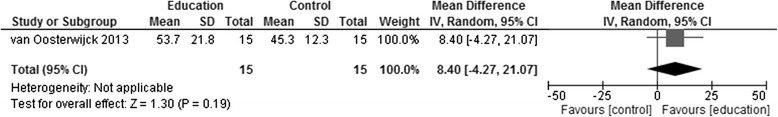
Forest plot showing disability (comparison of different types of education)—follow-up (3 months)

In the study [23] that contained a follow-up assessment (3 months after the intervention had ended), there was a mean difference of 8.4 points on the SF36 physical function subscale (range 0–100) in favour of PNE, which was not statistically significant (Fig. 7).

*Adults >65 years*: The one study exclusively investigating older adults (>65 years) used a very small sample size [17]. There were no significant differences between education about pain and education about physical methods to reduce pain either immediately after the intervention period or 6 weeks from its end.

3. Psychosocial outcomes (Table 6 post-intervention and Table 7 follow-up).

**Table 6:** Psychosocial outcome measures - post-intervention

Outcome measure	Study	Sample size			Statistic used	Heterogeneity	Effect size		Test for overall effect		Notes
		Intervention	Control	Total		*I* ^2^ (%)		[95 % CI]	*Z*-value	*p*-value	
CATASTROPHISING											
Education versus usual care											
CSQ – catastrophising PCP (EA) - catastrophising	Linton 1997; Soares 2002; Ruehlman 2012	219	185	404	SMD random	0	−0.08	[−0.28, 0.12]	0.79	0.43	Figure 8
Comparison of different types of education											
Pain catastrophising scale (PCS)	Moseley 2004; van Oosterwijck 2013; Gallagher 2013	86	81	167	SMD random	48	−0.81	[−1.27, −0.35]	3.47	0.0005	Figure 10; favours education
SELF-EFFICACY											
Education versus usual care											
CSQ - self efficacy	Soares 2002	18	17	35	MD random	n/a	0.47	[−0.83, 1.77]	0.71	0.48	
KNOWLEDGE OF PAIN											
Comparison of different types of education											
Pain biology/neuro-physiology knowledge	Gallagher 2013; van Oosterwijck 2013	55	54	109	MD random	0	3.86	[2.44, 5.28]	5.34	<0.00001	Figure 12; favours education
Knowledge and attitude score	Ferrell 1997	9	9	18	MD random	n/a	34.10	[23.22, 44.98]	6.14	<0.00001	Sample >65 years
GLOBAL HEALTH											
Comparison of different types of education											
SF36 - general health perceptions	van Oosterwijck 2013	15	15	30	MD random	n/a	−0.50	[−11.07, 10.07]	0.09	0.93	
SF36 - overall health rating	Ferrell 1997	10	10	20	MD random	n/a	−16.20	[−31.56, −0.84]	2.07	0.04	Favours control
MOOD											
Education versus usual care											
DASS – depression	Ruehlman 2012	162	143	305	MD random	n/a	−0.26	[−1.51, 0.99]	0.41	0.68	
Comparison of different types of education											
SF36 - mental health	van Oosterwijck 2013	15	15	30	MD random	n/a	13.40	[−1.24, 28.04]	1.79	0.07	
SOCIAL FUNCTION											
Comparison of different types of education											
SF36 – social function	van Oosterwijck 2013	15	15	30	MD random	n/a	8.90	[−8.16, 25.96]	1.02	0.31	

*SF-36* RAND 36-item health survey, *DASS* depression, anxiety and stress scale, *CSQ* coping strategies questionnaire, *PCS* pain catastrophising scale, *CSQ* coping strategies questionnaire, *PCP (EA)* profile of chronic pain (Extended Assessment), *95 % CI 95 %* confidence interval; Effect size represented as standardised mean difference (SMD) or mean difference (MD) depending on statistic used; Random = random effects model; heterogeneity is not applicable (n/a) when reported as single study

**Table 7:** Psychosocial outcome measures - follow-up

Outcome measure	Study	sample size			Statistic used	Heterogeneity	Effect size		Test for overall effect		Notes
		Intervention	Control	Total		*I* ^2^ (%)		[95 % CI]	*Z*-value	*p*-value	
CATASTROPHISING											
Education versus usual care											
CSQ – catastrophising PCP (EA) - catastrophising	Soares 2002; Ruehlman 2012	177	160	337	SMD random	0	−0.09	[−0.30, 0.13]	0.79	0.43	Figure 9
Comparison of different types of education											
Pain catastrophising scale (PCS)	van Oosterwijck 2013; Gallagher 2013	55	54	109	SMD random	0	−0.87	[−1.26, −0.47]	4.31	<0.0001	Figure 11; favours education
KNOWLEDGE OF PAIN											
comparison of different types of education											
Pain biology/neuro-physiology knowledge	Gallagher 2013; van Oosterwijck 2013	55	54	109	MD random	0	3.69	[2.22, 5.17]	4.90	<0.00001	Figure 13; favours education
knowledge and attitude score	Ferrell 1997	9	9	18	MD random	n/a	24.10	[9.15, 39.05]	3.16	0.002	Sample >65 years
GLOBAL HEALTH											
Comparison of different types of education											
SF36 - general health perceptions	van Oosterwijck 2013	15	15	30	MD random	n/a	9.10	[−1.07, 19.27]	1.75	0.08	
SF36 - overall health rating	Ferrell 1997	10	10	20	MD random	n/a	5.60	[−9.73, 20.93]	0.72	0.47	Sample >65 years
MOOD											
Education versus usual care											
DASS – depression	Ruehlman 2012	162	143	305	MD random	n/a	0.36	[−0.99, 1.71]	0.52	0.60	
Comparison of different types of education											
SF36 - mental health	van Oosterwijck 2013	15	15	30	MD random	n/a	18.20	[5.39, 31.01]	2.78	0.005	Favours education
SOCIAL FUNCTION											
Comparison of different types of education											
SF36 – social function	van Oosterwijck 2013	15	15	30	MD random	n/a	−3.10	[−19.13, 12.93]	0.38	0.70	

*SF-36* RAND 36-item health survey, *DASS* depression, anxiety and stress scale, *CSQ* coping strategies questionnaire, *PCS* pain catastrophising scale, *CSQ* coping strategies questionnaire, *PCP (EA)* profile of chronic pain (Extended Assessment); *95 % CI 95 %* confidence interval; Effect size represented as standardised mean difference (SMD) or mean difference (MD) depending on statistic used; Random = random effects model; heterogeneity is not applicable (n/a) when reported as single study

#### Education versus usual care

**Fig. 8:**
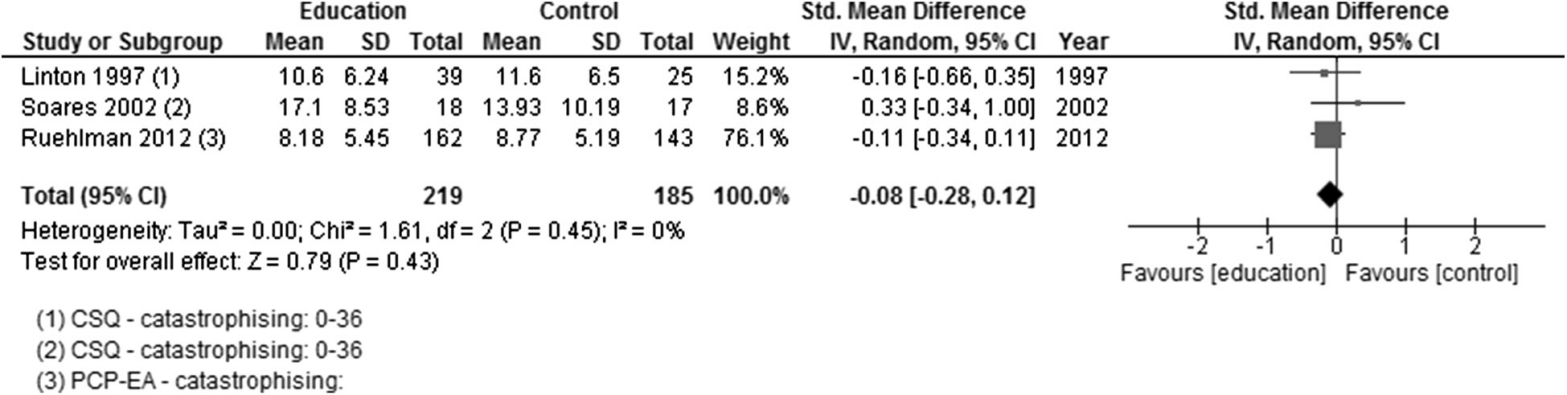
Forest plot showing catastrophising (education versus usual care)—post-intervention

**Fig. 9:**
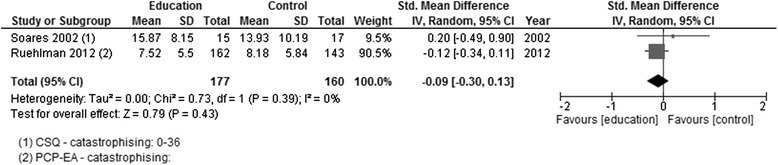
Forest plot showing catastrophising (education versus usual care)—follow-up (3 months)

*Adults >18 years*: Catastrophising was assessed in three of the four studies post-intervention [11, 20, 21]. Two had a follow-up assessment at 3 months from the end of the intervention [20, 21]. There was no effect, post-intervention, in any of the studies individually or when the data were pooled (Fig. 8), nor was there an effect at follow-up (Fig. 9). One study measured self-efficacy, only at post-intervention, and showed no effect [21]. Depression was measured in one study, and there were no changes post-intervention or at follow-up [20].

**Fig. 10:**
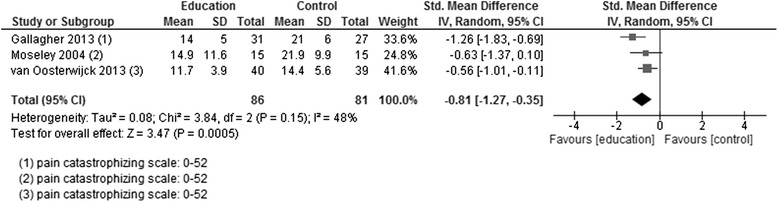
Forest plot showing catastrophising (comparison of different types of education)—post-intervention

**Fig. 11:**
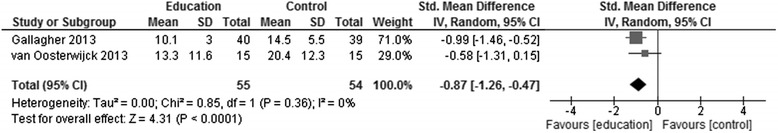
Forest plot showing catastrophising (comparison of different types of education)—follow-up (3 months)

**Fig. 12:**
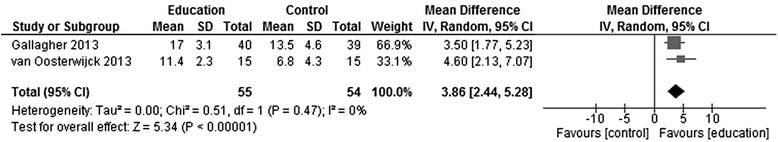
Forest plot showing knowledge of pain (comparison of different types of education)—post-intervention

**Fig. 13:**
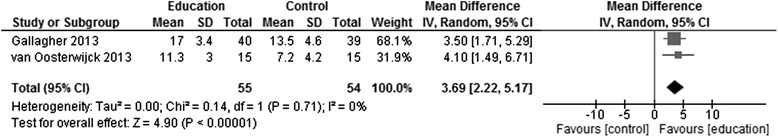
Forest plot showing knowledge of pain (comparison of different types of education)—follow-up (3 months)

*Adults >65 years*: One study carried out a bespoke measure of participants’ knowledge about pain and reported a significant improvement, post-intervention and at follow-up, in favour of the intervention [17].

#### Comparison of different types of education

*Adults >18 years*: Catastrophising was assessed in each of the three studies post-intervention [18, 19, 23], with two providing follow-up data [18, 23]. In each study and in the pooled data, there was a positive effect in favour of PNE at both assessment times (Fig. 6a, b). Two of the three studies assessed knowledge of pain post-intervention and at follow-up [18, 23]. A positive effect in pooled data in favour of PNE reflected the positive effects in both studies post-intervention and at follow-up (Fig. 7a, b). Only one of the studies assessed other relevant outcomes—mood, global health and social function [23]. For mood, there were positive effects post-intervention in favour of PNE, which did not reach statistical significance (*p* > 0.05) but did so at follow-up. There were no significant effects in global health or social function at either measurement point.

*Adults >65 years*: No specific data were available.

## Discussion

We systematically reviewed RCTs that investigated the effects of education to facilitate knowledge of chronic pain in adults on pain intensity and disability. Our analysis of the nine studies that fit the inclusion criteria found no evidence of an effect on pain intensity. However, for disability, there was evidence of a significant improvement immediately following a course of a particular type of education—pain neurophysiology education (PNE). Such an effect was not seen for the other types of education investigated in the studies.

Only one study specifically looked at people over 65 years old, also showing no significant effect on pain or disability.

Other reviews have been published in the past 5 years examining education for cancer pain [37], PNE for chronic musculoskeletal pain [36], PNE for chronic low back pain [35], education for neck pain [38, 39], educational interventions by pharmacists for chronic pain [40] and knowledge translation for chronic non-cancer pain management [41]. This last review included interventions aimed at health professionals, patients and a combination of target groups. Of these reviews, only three were able to combine studies to perform some meta-analyses within their reviews [35, 38, 40], whilst others reported results in the narrative.

### Pain severity/intensity

As in the current review, educational interventions had no significant impact on pain severity or intensity in whiplash-associated disorders (neck pain [38]) and no clinical significance in chronic low back pain [35], though it was shown to be significantly effective in reviews of education in cancer pain [37] and chronic musculoskeletal pain [38]. The variation in results with regards to the change (or lack thereof) in pain intensity may largely be due to the nature of the patient population (cancer patients [37]) or the intervention itself (multi-disciplinary approach combining education with physiotherapy or cognition-targeted motor control training [38]).

### Disability/physical function

Other reviews [35, 38] have not revealed significant change in levels of disability and function, consistent with the present review.

Conversely, Louw et al. [38] described a significant effect from education in those with musculoskeletal pain. Included in that review [38] was the one study in the present review that showed a significant improvement in disability as a result of the intervention [19], and others by the same research team (five out of eight trials), potentially skewing the results of the review to reflect this one intervention. The review of knowledge translations targeting patients showed short-term improvements in patient function with chronic low back pain, but no change in migraine-related complaints [41], suggesting as we have in the inclusion and exclusion criteria of the present review that underlying conditions (such as migraine) should be treated and analysed separately to other manifestations of chronic pain.

### Psychosocial outcomes

The most interesting findings were the significant improvements in catastrophising and knowledge of pain. The improvements in catastrophising were only found in those studies that utilised pain neurophysiological education (PNE) in the intervention. This fits with one of the primary aims of PNE, to reconceptualise thinking about pain, away from the belief that “hurt” always equates to “physical harm”. The change in knowledge about pain, which were also seen with PNE, also point towards achievement of this primary aim. However, the design of the studies and reliance on questionnaire findings do not allow the depth of investigation needed to fully explore this suggestion, and appropriate qualitative investigation is called for. Interestingly, knowledge of pain was found to increase in the single study on older people exclusively [17] (not using PNE). This suggests that such an aim (to reconceptualise thinking) is not limited by older age. However, the measurement used in the study was very superficial, and again, the most appropriate action would be to explore this in more depth.

This review was conducted using the most robust techniques available. Electronic searches included full access to four databases (MEDLINE, CINAHL Plus, EMBASE and CENTRAL [Cochrane Central Register of Controlled Trials]), alongside international trial registries and author personal libraries. In total, 8519 titles were assessed for inclusion (8371 of published papers, 148 from ongoing trial registry). The review summarises the highest quality evidence available using RCTs of reasonable quality. The use of meta-analytical methods to pool data from different studies, which had relatively small individual sample sizes, maximised the strength of the findings.

The search was undertaken from database inception until 31 December 2013, and all data were extracted and analysed within 6 months of this date. The resources available prevent us from updating and re-analysing the search, and we are unable to examine the effect that any subsequent studies may have on our findings. This is an area for ongoing research, which will be supported by our included list of ongoing trials noted at the time of our analysis.

The review was limited by the small number of studies suitable for analysis, reflecting the availability of relevant published studies. This meant that findings were based on relatively low sample sizes, although this was overcome to an extent by pooling of data where appropriate. Because of the small numbers available, we were not able to carry out subgroup analyses to assess the influence of study quality on outcomes nor were we able to comprehensively assess the influence of older age.

The scope of the review was deliberately restricted to investigate education in isolation from other interventions, and care should therefore be taken when extrapolating the findings to the use of education delivered in combination with other interventions. However, there is room for future study of the additive effects of education in combination with other approaches to pain management.

We also used outcomes of pain intensity and disability as inclusion criteria. Therefore, in our post-hoc analysis of psychosocial outcomes, it is likely that we have excluded evidence from studies that used such measures but did not measure pain or disability; other sources of evidence should be used to make judgement on the effect of education on other outcomes such as mood, coping strategies and pain beliefs, all of which can be important in pain management.

We did not contact authors for further information and excluded papers that were not available in English at the full-text stage only. Both of these decisions were largely made as a result of the resources available to us. However, the need to contact authors only occurred in the case of a single paper [12], where details were lacking regarding group sample sizes, and no variation around the mean was reported. We decided we were unlikely to receive a response due to the considerable time period since publication (1988), and as a result, the paper was excluded from the meta-analyses. Only one paper [42] was excluded due to the language (Fig. 1), and we are unable to judge the effect of this exclusion on our results.

As highlighted by the small number of studies and the diverse range of educational methods that have been used in the current literature base, there is a general need for more high quality trials in this area. The specific findings for PNE, in this review and in others, should stimulate research to see how its promise can be optimised to further improve its effects, perhaps by comparing different methods of delivery and tailoring its content to specific populations including older people.

One study [28] compared two different modes of delivery (website versus written material), though it was excluded due to the large cognitive behavioural therapy (CBT) component delivered through the website. Equivalent research examining mode of delivery would be of interest to further examine whether online delivery of an educational intervention, for example, is equally effective across all age groups or whether it is the group element compared to individual learning that has the greatest influence on effect size.

The wide variety of assessments available for this review meant that a great deal of analysis was not possible due to the variability of the focus of the assessments or when subscales were not reported. Future meta-analyses could be improved through the standardisation of outcome measures.

Further, the effect of education on psychosocial variables as mediators of pain and disability remains to be elucidated. Research to investigate the effect of education upon knowledge and its relationship to psychosocial mediators is warranted.

Finally, a long-term follow-up should be implemented for all studies, as short-term results whilst promising, do not necessarily suggest long-term effect.

## Conclusions

Of the different forms of education reported in RCTs, only PNE appears to be effective (by reducing disability) as a sole intervention for adults with chronic pain and only immediately after the intervention. However, the evidence is too limited to conclusively rule out other options.

### Practical implications

Education to facilitate knowledge of chronic pain in adults remains a potentially important part of patient activation. Certainly, for people with established chronic pain (as represented by the studies reviewed here), it would be sensible to include education along with other interventions as there is little evidence to support education as a stand-alone intervention. Whilst research findings continue to emerge, clinicians should consider incorporating PNE, though it would be premature to discard other options. However, we cannot confidently conclude that education alone is effective in reducing pain intensity or related disability in chronic pain in adults.

## Additional files

Additional file 1:
**Search strategy.** (DOCX 13 kb) Additional file 2:
**Ongoing (unpublished) trials.** (DOCX 16 kb)

## Abbreviations

CBT: cognitive behavioural therapy
CI: confidence interval
GP: general practitioner
ICTRP: International Clinical Trial Registry Platform
MD: mean difference
MPQ: McGill Pain Questionnaire
PNE: pain neurophysiology education
RCT: randomised controlled trial
RMDQ: Roland Morris Disability Questionnaire
SD: standard deviation
SE: standard error
SMD: standardised mean difference
TENS: transcutaneous electrical nerve stimulation
VAS: visual analogue scores
WHO: World Health Organisation
