# Supplementary material for: Effects of education to facilitate knowledge about chronic pain for adults: a systematic review with meta-analysis
Source: Syst Rev. 2015 Oct 1;4:132. doi: 10.1186/s13643-015-0120-5 (PMC4591560; doi:10.1186/s13643-015-0120-5)
Supplement: Additional file 1: — Search strategy. (DOCX 13 kb) [file 13643_2015_120_MOESM1_ESM.docx]

**APPENDIX 1 – SEARCH STRATEGY**

1. Self Care/

2. (self adj3 (manag* or care)).tw.

3. Self-Help Groups/

4. (self-help or "self help").tw.

5. selfhelp.tw.

6. Patient Education as Topic/

7. ((patient* or adult* or client* or participant* or individual*) adj3 (train* or educat* or teach* or instruct* or inform* or counsel* or empower* or advic*)).tw.

8. (bibliotherapy OR learn* OR guid* OR facilitat*)

9. or/1-8

10. exp Pain/

11. pain.tw.

12. 10 or 11

13. randomized controlled trial.pt.

14. controlled clinical trial.pt.

15. randomized.ab.

16. placebo.ab.

17. drug therapy.fs.

18. randomly.ab.

19. trial.ab.

20. or/13-19

21. exp animals/ not humans.sh.

22. 20 not 21

23. 9 and 12 and 22
