# Supplementary material for: Effects of education to facilitate knowledge about chronic pain for adults: a systematic review with meta-analysis
Source: Syst Rev. 2015 Oct 1;4:132. doi: 10.1186/s13643-015-0120-5 (PMC4591560; doi:10.1186/s13643-015-0120-5)
Supplement: Additional file 2: — Ongoing (unpublished) trials. (DOCX 16 kb) [file 13643_2015_120_MOESM2_ESM.docx]

**APPENDIX 2 – ONGOING TRIALS (UNPUBLISHED)**

| **Study ID, date registered (dd/mm/yyyy)** | **Public title/ registered title** | **Methodological quality** | **Population** | **Interventions** | **Primary outcome** | **Other Outcomes** |
| --- | --- | --- | --- | --- | --- | --- |
| ACTRN12613000730707  (02/07/2013)  not yet recruiting | The role of education in the self-management of elderly patients with persistent (chronic) pain | permutated block randomisation by online random number generation, RCT, no masking (open label) | age>60yrs, chronic pain (>3months), independent, newly referred to pain clinic | 5hrs of education over 2 sessions in one week. | Pain management Strategies Questionnaire at baseline, 2wks, and 3months | pain self-efficacy questionnaire (PSEQ), Brief Pain Inventory (BPI), Global perceived impression of changes (GPIC), patient satisfaction. At 2wks and 3months post-intervention |
| NCT01794988  (15/02/2013)  recruiting | Can therapy alter CNS processing of chronic pain: a longitudinal study | randomised, RCT | age 18-75yrs. Chronic pain for 12months (musculoskeletal, non-neuropathic) | 4 arms (3 intervention, 1 control): relevant intervention "pain education" for 11wks - mailed education to home address on weekly basis, others "groupCBT" for 11wks, "therapeutic interactive voice response (TIVR)" for 4months | Pain | none listed |
| ISRCTN67153366 (29/09/2006)  Completed - no publications found | Promoting a self-care approach for managing persistent pain | RCT, computer random selection tool, blinding not possible | age 18-75yrs. Chronic pain (>3months), referred to pain management service, literate | 6wk, 2.5hr/wk, self-care programme "Expert Patient Programme" (EPP) | Pain stage of change questionnaire (PSOQ) at baseline, 4 and 9months | none listed |
| NCT00224991  (21/09/2005)  Completed - no publications found | Osteoporosis school | RCT, no masking (open label), | age>50yrs. Fracture due to osteoporosis | "intensive systematic information" (osteoporosis school) | fall frequency | compliance, quality of life |
| ISRCTN53460881 (08/11/2012)  Completed - no publications found | Self-management migraine Headache Education (SHE) | randomised, RCT | age 18-75yrs. Chronic migraine (6months, on more than 4days in past month), English speaking | SHE: 3 fortnightly one-to-one sessions with nurse plus 2 telephone calls between. Each session 50mins, each phone call 10-20mins. | headache frequency (days/month) at 8wks and 4months | headache impact (HIT-6) at 8wks and 4months, health costs, migraine diability (MIDAS) at 8wks and 4months, headache related beliefs (illness perception questionaire-revised) at 8wks and 4months, psychological morbidity (HADS) at 8wks and 4months |
| NCT00417742  (02/01/2007)  Completed - no publications found | Project CHEER (Comprehensive Headache Evaluation, Education, Relief) | random allocation, no masking (open label), RCT | Age>21yrs. Chronic headache (tension type headache, migraine, or mixed etiology), MIDAS>5. | single intervention "Education": service delivery through a Headache Management Program | MIDAS questionnaire | CATI headache diagnostic survey (baseline), SF36 QoL (Baseline, 6 and 12months), Healthcare utilisation measures (self-report; baseline, 3, 6, 12 months), use of alternative therapies (self-report) |
